# Bioimpedance Sensor and Methodology for Acute Pain Monitoring

**DOI:** 10.3390/s20236765

**Published:** 2020-11-26

**Authors:** Mihaela Ghita, Martine Neckebroek, Jasper Juchem, Dana Copot, Cristina I. Muresan, Clara M. Ionescu

**Affiliations:** 1 Research Group of Dynamical Systems and Control, Ghent University, Tech Lane Science Park 125, 9052 Ghent, Belgium; Jasper.Juchem@UGent.Be (J.J.); Dana.Copot@UGent.Be (D.C.); ClaraMihaela.Ionescu@UGent.Be (C.M.I.); 2 EEDT—Core Lab on Decision and Control, Flanders Make Consortium, Tech Lane Science Park 131, 9052 Ghent, Belgium; 3 Department of Anesthesia, Ghent University Hospital, C. Heymanslaan 10, 9000 Gent, Belgium; Martine.Neckebroek@UGent.Be; 4 Department of Automation, Technical University of Cluj-Napoca, Memorandumului 28, 400114 Cluj-Napoca, Romania; Cristina.Muresan@aut.utcluj.ro

**Keywords:** noninvasive pain sensor, electrical impedance spectroscopy, time–frequency analysis, model identification, fractional-order impedance model, nociceptive stimulation

## Abstract

The paper aims to revive the interest in bioimpedance analysis for pain studies in communicating and non-communicating (anesthetized) individuals for monitoring purpose. The plea for exploitation of full potential offered by the complex (bio)impedance measurement is emphasized through theoretical and experimental analysis. A non-invasive, low-cost reliable sensor to measure skin impedance is designed with off-the-shelf components. This is a second generation prototype for pain detection, quantification, and modeling, with the objective to be used in fully anesthetized patients undergoing surgery. The 2D and 3D time–frequency, multi-frequency evaluation of impedance data is based on broadly available signal processing tools. Furthermore, fractional-order impedance models are implied to provide an indication of change in tissue dynamics correlated with absence/presence of nociceptor stimulation. The unique features of the proposed sensor enhancements are described and illustrated here based on mechanical and thermal tests and further reinforced with previous studies from our first generation prototype.

## 1. Introduction

Human bioimpedance allows for the characterization of pain pathway by providing an electrical signature, whose time and frequency modulation informs on physiological and electrochemical phenomena. Beside healthcare assessment systems, skin impedance has been studied in multiple medical applications as an indicator for the functioning of the human body [1]. There is a wide spectrum of its utilization for cancer analysis [2], changes in the physiological state, virus detection [3], or sensation of fear [4]. Nowadays, bioimpedance is increasingly used to estimate the body composition [5,6] and to interpret the histological composition of different tissues [7].

Electrode recordings of sympathetic activity in skin nerves have been correlated with blood pressure and electrodermal activity (EDA) even from early times. EDA has a long history in psycho-physiological research, when observations showed a connection between skin electrical activity and mental stress, sweat glands, or pain [8]. Despite the common acceptation of its significance, the researchers’ interest for EDA in pathophysiological applications was revived recently [9]. Investigation of EDA measurements has been increasingly used for quantitative assessment of nociception, as pain monitoring remains a well-known problem in clinical management [10]. Nearly 20% of patients still experience severe postoperative pain and approximately 10% have chronic post-surgical pain [11], while intraoperative awareness continues to occur with reported pain perception [12].

This paper aims to raise awareness in exploring the full potential of using bioimpedance spectroscopy for the monitoring of pain. Nowadays, several such devices are available, including electrodermal response analysis through variability in electrophysical properties [13] and multivariate signal analysis [14] in time domain. An overview of commercialized nociception monitors is depicted in [15]. However, the existing reported evidence on the pain monitors from the clinical trials remains insufficient for their broad applicability and inconclusive [15,16,17]. Besides, inadequate or subjective pain assessment can cause complications, so costly errors can be minimized and patient safety maximized by using a pain monitor. Another reason for such a monitor is the economic burden of pain management that requires sensing solutions for pain in an objective and non-invasive manner.

The paper presents an analysis of the relationship between pain and dermal impedance with an in-house developed prototype adequately approved for clinical testing. Mainly, the development and performance-analysis of Anspec-PRO sensing device—second generation—are addressed. Anspec-PRO differentiates from the commercial devices in exploring the full potential of bioimpedance data: 2D to 3D time–frequency analysis. The novelty of this approach consists in using a multi-frequency excitation signal for pain detection, instead of a fixed frequency value which delivers a single point of analysis in time–frequency domain; hence, we obtain a surface or spectrogram, broader in information for a full characterization of the biological tissue response. Using the image-based results, the application of artificial intelligence algorithms can further contribute in predicting the pain response. Moreover, the possibility to explore skin excitation by different self-customized signals and protocols adds value in research. Based on the availability of the input and output signals, a transfer function can be provided for mathematical modeling. This additional original element was shown by the development of the parametric nociceptor model [18].

The prototype Anspec-PRO was validated on awake healthy volunteers (part of the development team) undergoing induced pain, with successful results in detecting and characterizing perception of pain [19]. Beside tests within controlled laboratory environments, the second generation device was clinically validated by means of a comparison with a reference commercial device, both methodologies based on electrodermal activity identification. The observational study in awake postoperative patients concluded that Anspec-PRO performs equally well as the commercial device under similar conditions, representing proof of concept for the prototype device [20]. Moreover, Anspec-PRO is currently under tests in a clinical trial on anesthetized patients for its validation in surgery context, in comparison to other two commercial pain monitors. The aim of the trial is also to develop an innovative pharmacodynamic model that can directly link the effects of opioid to an objective pain index.

Here, the detailed hardware and software components of Anspec-PRO along with a signal analysis are given as follows. Section 2 highlights the methodologies for bioimpedance data analysis and multisine design. Next, the device is tested in various conditions and the results discussed. The Discussion Section 4 summarizes the main advantages of time–frequency bioimpedance for pain evaluation and underlines the versatility of Anspec-PRO device. The paper ends with conclusions and future perspectives.

## 2. Materials and Methods

### 2.1. Background

The last decade reported various efforts in updating and increasing performance of bioimpedance spectroscopy analyzers, with multi-disciplinary expertise from medical, biological, and engineering areas. As accuracy increases, the complexity of these non-commercial devices tends to increase as well [21,22]. However, the medical practitioner prefers user-friendly devices [23]. The disadvantage of the end-user devices is that they are relatively expensive, non-transparent, and often limited in versatility. The medical practitioner needs to make a decision in selection of analgesia devices from a myriad of methodologies. Consequently, many engineering tools tend to miss the transfer to the medical research labs as they are too complex to be understood.

Bioimpedance is expressed as a complex number, which is frequency- as well as time-dependent Z(ω,t)∈C[24]. It is a dependent variable that is described by the angular frequency ω (rad/s) and time *t* (s). Explored at its full potential, it allows monitoring changes in both time and frequency domain. By using electrical analogy for skin impedance, it is defined as the ratio of voltage over current in frequency domain.

In other commercially-available devices, the variability of the phase shift as a function of frequency is neglected. Hence, the potential of data gathering at no extra cost is not explored. For instance, Bodystat™Multiscan 5000 and Impedimed™L-Dex U400 use a fixed frequency range as these are focused on a single application step. The MedStorm™PainSensor uses the information of a single frequency that is fixed and evaluates time-domain variability of the conductance (i.e., the real part of admittance). Most of these devices do not allow exporting the raw data for further analysis and often output surrogate variable parameters (e.g., L-Dex value, PainMonitor Index, etc.). No claims can be made on the identification accuracy, making it difficult (if not impossible) to adequately compare these tools. These commercial devices perform well in specific case for clinical monitoring, but not for research purposes.

**Figure 1:**
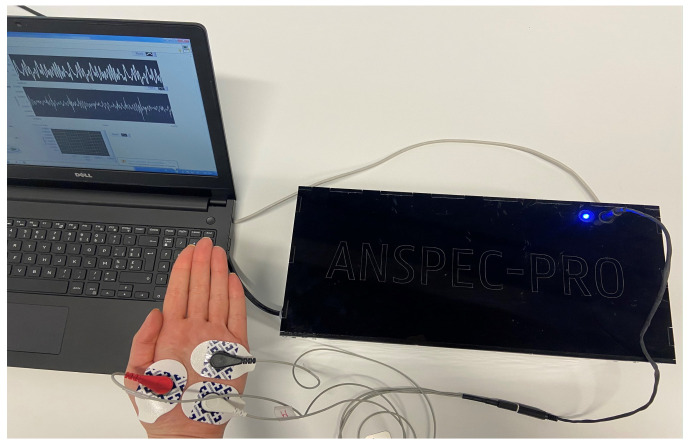
The second generation ANSPEC-PRO prototype and related set-up for non-invasive measurement of skin impedance.

This paper provides a second generation prototype that can be tested with different protocols or self-designed excitation signals, allowing a complex analysis and modeling of skin biological phenomena. The tested hypothesis is that skin frequency response provides a detection mechanism and enables modeling of nociception process (i.e., that is dynamic). This rationale is justified by the fact that molecular levels are excited, hence generating the action potential which is in charge with autonomic response and ionic diffusion. The second generation device developed in our laboratory, which has already received approval to be used in clinical trials as a medical device, is depicted together with the measurement set-up in Figure 1. Three electrodes are placed on the palmar skin: two current-carrying (white and red) and one pick-up electrode that measures only the voltage (black); hence, no polarization occurs. The hardware and software specifications, as well as the user interface for measurements settings, are detailed in Appendix B, Appendix C andAppendix D for both device generations.

### 2.2. Multisine Data-Analysis

**Figure 2:**
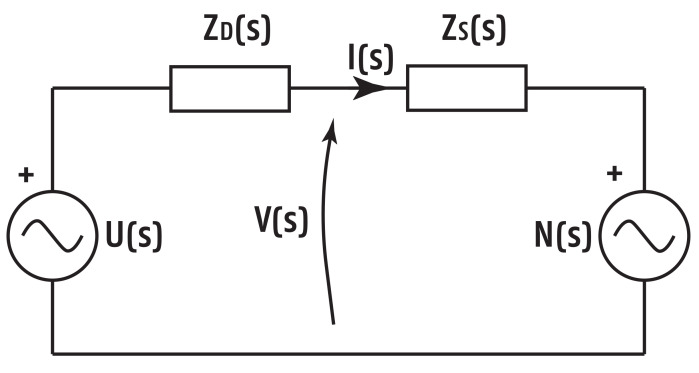
Electrical analogy scheme for skin impedance with nociception stimuli.

Following impedance modeling analogy from prior work [25,26], the electrical analogy is made as in Figure 2. The following variables are defined: U(s) is the off-line designed input/excitatory multisine signal, $Z_{D}\left(s\right)$ is the impedance of the device itself (known), $Z_{s}\left(s\right)$ is the skin impedance (unknown), N(s) is the unknown secondary input as nociceptor stimulus, V(s) is the actual voltage over the skin impedance, and I(s) is the current running through the skin. As observed, the system has two inputs: the known multisine excitation signal U(s) and the unknown nociceptor stimulus N(s). The measured voltage and current signals, V(s) and I(s), respectively, are the outputs. The variable *s* denotes the Laplace operator.

The analysis transforms the two time vectors to an impedance in frequency domain using the classical periodogram method by replacing s⟶jω, with $j=\sqrt{-1}$ and ω=2πf (rad/s) with *f* frequency (Hz). We have that:

(1) $$\begin{array}{cc} V(s) & =\frac{Z_{s}\left(s\right)}{Z_{D}\left(s\right)+Z_{s}\left(s\right)}U\left(s\right)+\frac{Z_{D}\left(s\right)}{Z_{D}\left(s\right)+Z_{s}\left(s\right)}N\left(s\right) \end{array}$$

(2) $$\begin{array}{cc} I(s) & =\frac{1}{Z_{D}\left(s\right)+Z_{s}\left(s\right)}U\left(s\right)-\frac{1}{Z_{D}\left(s\right)+Z_{s}\left(s\right)}N\left(s\right) \end{array}$$

or equivalent in matrix form:

(3) $$\left[\begin{array}{c} V(s)\\ I(s) \end{array}\right]=\underline{Z}\left(s\right)\left[\begin{array}{c} U(s)\\ N(s) \end{array}\right]$$

with

(4) $$\underline{Z(s)}=\frac{1}{Z_{D}\left(s\right)+Z_{s}\left(s\right)}\left[\begin{array}{cc} Z_{s} & Z_{D}\\ 1 & -1 \end{array}\right]$$

Fourier transform gives the corresponding cross- and auto-power spectra:

(5) $${\underline{S}}_{YU}\left(j\omega \right)=\left[\begin{array}{c} S_{VU}\left(j\omega \right)\\ S_{IU}\left(j\omega \right) \end{array}\right],{\underline{S}}_{UU}\left(j\omega \right)=\left[\begin{array}{c} S_{UU}\left(j\omega \right)\\ S_{NU}\left(j\omega \right) \end{array}\right]$$

It follows that:

(6) $${\underline{S}}_{YU}\left(j\omega \right)=\underline{Z}\left(j\omega \right){\underline{S}}_{UU}\left(j\omega \right)$$

In the absence of a nociceptor stimulus (N(s)=0), or if the stimulus is uncorrelated to the input, the multisine signal (6) reduces to:

(7) $$\left[\begin{array}{c} S_{VU}\left(j\omega \right)\\ S_{IU}\left(j\omega \right) \end{array}\right]=\frac{1}{Z_{D}\left(j\omega \right)+Z_{s}\left(j\omega \right)}\left[\begin{array}{c} Z_{s}\left(j\omega \right)\\ 1 \end{array}\right]S_{UU}\left(j\omega \right)$$

from which the impedance of interest can be estimated:

(8) $${\hat{Z}}_{s}\left(j\omega \right)=\frac{S_{VU}\left(j\omega \right)}{S_{IU}\left(j\omega \right)}$$

For identification of ${\hat{Z}}_{s}\left(j\omega \right)$, the nociceptor signal acts as a *disturbance*. By designing the input signal U(s) such that it is uncorrelated with the nociceptor stimulus ($S_{UN}\equiv 0$), (8) holds.

### 2.3. Multisine Design

To excite multiple frequencies at the same time, a superposition of sines with different frequencies can be used: a multisine. An advantage of this excitation signal is that one can measure a broad frequency band in a relatively short time-frame, which is useful in fast time-varying systems. The multisine signal has the following formulation:

(9) $$V_{in}\left(t\right)=\sum_{i=1}^{l}A_{i}\sin\left(2\pi f_{i}t+\phi_{i}\right)$$

with frequency $f_{i}=f_{0}+\frac{f_{l}-f_{0}}{l}i$ (i=0,1⋯l) and $\phi_{i}\in \left[0,2\pi \right]$ uniformly distributed pseudo-random number, giving the sine a random phase shift. Because of the random character of the phase shifts, the crest factor is not optimized. The crest factor indicates the compactness of the signal and is given as $u_{peak}/u_{RMS}$, with $u_{peak}$ the peak value of the signal and $u_{RMS}$ the Root Mean Square of the signal in the frequency band of interest. A lower crest factor means that more power is injected into the system [27].

For the experiments presented in this paper, an off-line odd multisine is designed, as detailed in [27], with number of samples N=300. To avoid spectral leakage, the measurement time is an integer number times the period of the multisine signal. The length of one period is $M_{s}=0.02$ s, resulting in a sampling frequency $f_{s}=15$ kHz. The input signal provides a persistent excitation for identification of the skin impedance within the $\left[f_{0};f_{l}\right]=\left[100;1500\right]$ Hz frequency interval and has 29 frequency points within these boundary values, linearly distributed in steps of 50 Hz.

Frequency response function (FRF) of a dynamical system is a measure of the modulus and phase of the output signal as a function of an input frequency, relative to the input signal applied to the system. FRF provides approximate models for the unknown process. To appropriately characterize the process dynamics in a given frequency interval, the gathered information must cover the modulus (in dB) and the phase (in radians or in degrees), or, equivalently, the complex impedance in real and imaginary parts as a function of frequency (in rad/s or in Hz). Classical methods for estimating FRF are based on input and output data followed by Fast Fourier Transform (FFT). To estimate the dynamical frequency response over the frequency range, the process needs to be excited with input signals of the desired frequencies. Therefore, Anspec-PRO is used for skin excitation over the frequencies specific for the response of tissue molecules involved in the nociception process.

From the electrodermal activity viewpoint, the activity of the skin layer where the sweat glands are located (i.e., the stratum corneum) is typically dominated by measurements at low frequencies (below 1 kHz), while viable skin dominates at higher frequencies (above 100 kHz) [24]. On the other side, of interest are also the nociceptors activity and action potentials that are triggered by the painful stimulus, so the whole skin is of interest, from epidermis to deeper layers of dermis. The free sensory nerve endings can be traced deeper into the epidermal layer or into dermis [28], and, because measurement depth is strongly dependent on excited frequency range, measurements done at higher frequencies will capture a bigger skin volume [24].

From the electrical permittivity viewpoint, there are described three different dispersion regions for biological materials, as follows: α, 10 Hz–10 kHz, for counter-ion effects, active cell membrane effects, gated channels, ionic diffusion (extracellular fluid level), etc.; β, 1 kHz–100 MHz, for dielectric property measurement of the cell membrane, passive cell membrane capacitance, etc.; and γ, >10 MHz, for content measurement of the biological species (intracellular fluid level), etc. [24,29,30]. Since pain is an electrical signal transmitted by dynamics of potassium channels signaling among intra- and extracellular fluid, it seems natural to choose the lowest frequency dispersion for exciting the skin with our device. Concluding, while the stratum corneum with sweat gland activity has been shown to display a very broad α dispersion, viable skin has electrical properties that display separate α and β dispersions [24]. Nevertheless, these different dispersions make the electrical properties converge as the frequency is increased.

However, the frequencies depend on the applicability of the bioimpedance analyzer, so may differ for skin moisture or ischemia level [31], specific cells detection [32,33], muscles assessment [34], and human body condition analysis [35,36,37]. In short, the different frequencies at which these devices excite the skin are summarized in Appendix A. Usually, bioimpedance measurements for electrodermal activity is done by each device over a wide range of frequencies up to 1 MHz, typically at 50 kHz [1,38]. Following this, the present work applies the existing *know how* of devices for impedance spectroscopy, but customized for pain measurement. The results included in this paper show the capability of Anspec-PRO to detect pain through skin measurements for the indicated frequency range.

To conclude, unlike commercial devices, Anspec-PRO is not limited to application of a single sinusoidal signal and it is versatile to user-defined multisine design.

## 3. Results of Experimental Validation

### 3.1. Known Impedance Values

To validate the accuracy of the Anspec-PRO device, a known probe electric circuit is measured. The multisine, as discussed in the previous section, with an amplitude of 5 V is used as input signal. For this analysis, a measurement time of 60 s was chosen. The probe circuit consists of a series connection of a resistor $R_{2}=12$ kΩ and capacitance $C_{1}=15$ nF with a parallel resistor $R_{1}=10$ kΩ. This circuit has the following transfer function model:

(10) $$Z_{RC}\left(s\right)=\frac{1.84s+9968}{3.386\cdot 10^{-4}s+1}$$

**Figure 3:**
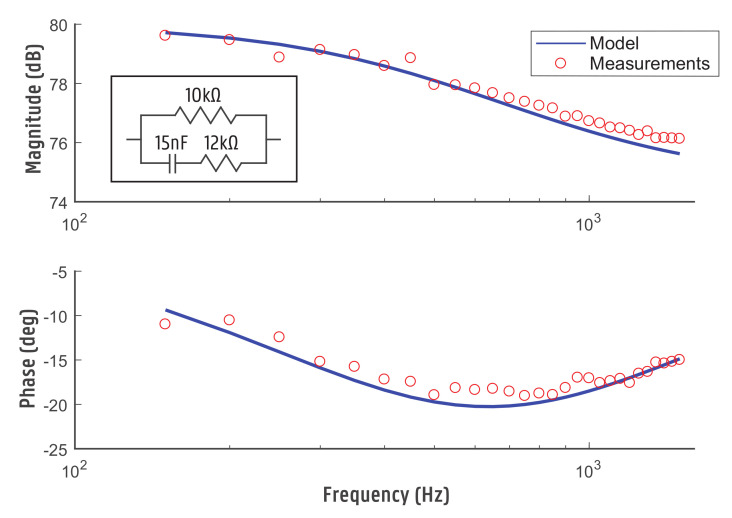
Bode plot of a measured and modeled RC test circuit.

**Figure 4:**
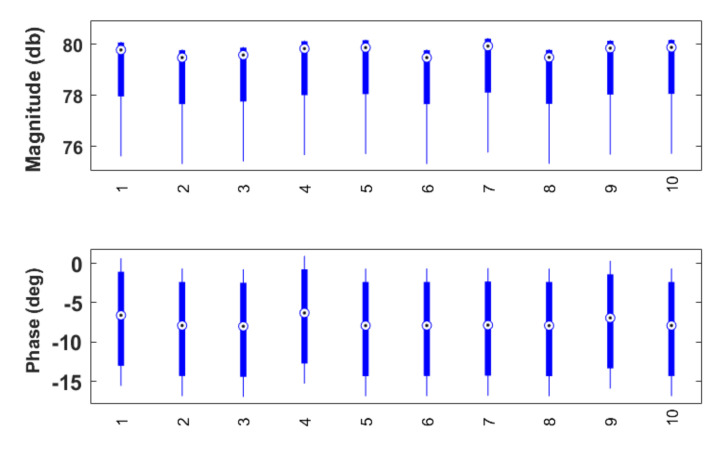
Boxplot analysis of the set of measurements performed to validate the accuracy of the developed device.

Ten consecutive measurements are performed on this sample circuit with the statistical analysis depicted in Figure 3 and Figure 4. For the magnitude, the %MSRE is 0.4% and for the phase the %MSRE is 7.2%. An accurate magnitude is obtained, whereas the error for the phase shows to be larger. The low variability for consecutive experiments (standard error of 1.3946 Ω and 0.1331${}^{\circ }$ for the modulus and phase respectively) shows that the device is trustworthy.

The obtained results suggest the device is reliable and has a good accuracy and resolution for the chosen frequency interval. It should be noticed that the circuit is not designed to evaluate any impedance circuits: large phase shifts can lead to instability in the Voltage Buffer circuit. Following this, the Op-Amp is not able to drive the load and the results are inconsistent. For our bioimpedance spectrum analysis applications, the phase shift never reaches the unstable region.

### 3.2. First Hand Results

The location of the electrodes is decided for the palmar skin, because increased sweat gland activity, as well as generation of action potentials elicited by nociceptor membrane activation, reveal specific reactivity to psychological stimulation at palmar sites due to the greater density on these areas. However, electrode placement is a generic problem and is discussed as a function of the application area [39]. As to the accuracy of the impedance values between the absence and presence of pain stimulus, sensors location should be fixed during the protocol and during clinical trials when compared among cohort groups.

As a proof of concept experiment, the claim is that changes occur in skin impedance during stimulation, i.e., when perceived as pain in one awake healthy volunteer. This was investigated on one of the manufacturers, age 35, height 171 cm, weight 83 kg, female, measured for five time intervals. An input multisine provides a persistent excitation to the skin within the frequency range of 10–77 Hz, while alternating absence and presence of stimulation generated by mechanical pressure on the finger.

The protocol is as follows:

- Minutes 1–5: The subject is in the nominal state, without applying any pain stimulation (NP1).
- Minute 6: Mechanical pain stimulation is applied by gripping the subject’s finger. The pain test is known as pressure test (P1).
- Minutes 7–9: The pain stimulation is ceased (NP2).
- Minutes 10–11: Mechanical pain stimulation is continuously applied as earlier (P2).
- Minutes 12–15: The pain stimulation is ceased (NP3).

**Figure 5:**
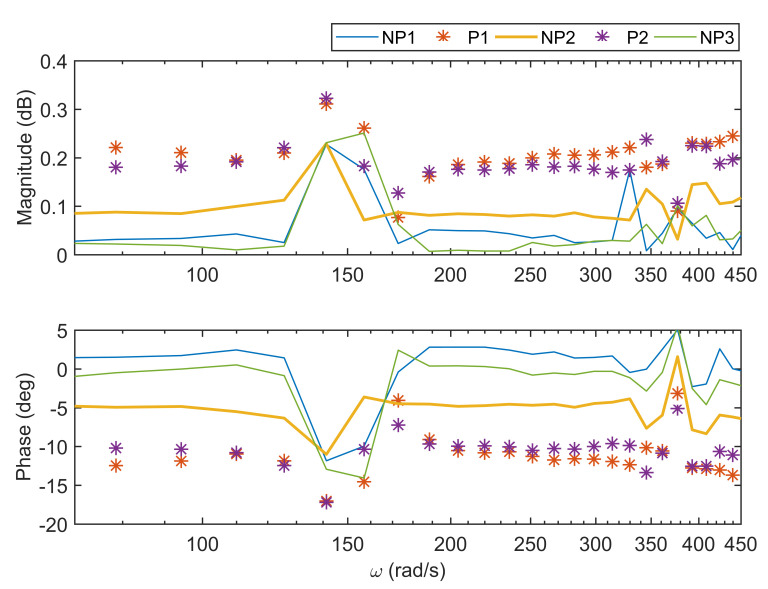
The Bode plot of the measured impedance for one volunteer during pressure test (NP denotes no pain and P denotes pain).

To show the reliability of the device, the measured impedance for one subject is given in Figure 5 for all measured intervals. From the Bode plot of the normalized complex impedance, it can be seen that the investigated hypothesis is validated, where the magnitude corresponding to pain intervals (i.e., P1 and P2) differentiates from absence of pain (i.e., NP1, NP2, and NP3). Additionally, the phase constancy is presented for all intervals. The rationale is based on the transmission in signaling pathways that occurs via neuronal activity, namely a framework of cascaded processes, which can be simplified to that of a ladder network. A constant-phase has already been revealed for neural populations, modeled as resistance-inductance equivalent electrical network recurrent elements with such collective behavior [40]. We can speculate that the constant phase for the pain intervals has an average of −10${}^{\circ }$, which suggests a fractional order 0.1, while the magnitude possibly has 2 dB for decade, as the slope seems to be 0 (i.e., magnitude has a constant of about 0.2 dB).

**Figure 6:**
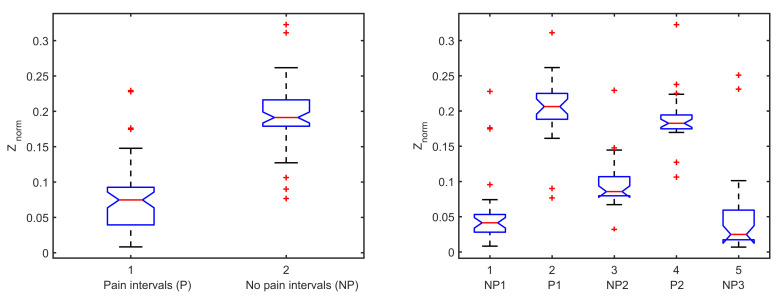
Statistical analysis of the measured bioimpedance for one volunteer during pressure test.

From the analysis of variances presented in Figure 6, the mean for pain intervals (P) is statistically different from no pain intervals (NP), with *p*-value <0.5. The mean of impedance values for each excited frequency differs significantly between NP1, P1, NP2, P2, and NP3, respectively.

### 3.3. 3D Representation of Impedance

Time–frequency analysis tests the hypothesis that the skin impedance is a dynamic system, where its dynamic properties are caused by the noxious stimulation. To characterize this black-box-like process, the need of a mathematical model is obvious. The theory of system engineering regarding identification states that a continuous excitation at system’s specific frequencies is needed [27]. In this way, we are able to extract a transfer function of the process, which describes the dynamics taking place. While a transfer function cannot be estimated in a single frequency point, Anspec-PRO sends a multisine signal composed of sum of sinusoids in a band-limited frequency interval.

A multisine with amplitude 5 V is used, giving the impedance Z(ω) for 29 frequencies in the [100,1500] Hz calculated protocol interval. In biological systems, the acquired signals may have components from the high-frequent noise of the same order of magnitude. In our case, the complex impedance topology of the skin tissue is reproducible without the design of complex filtering.

**Figure 7:**
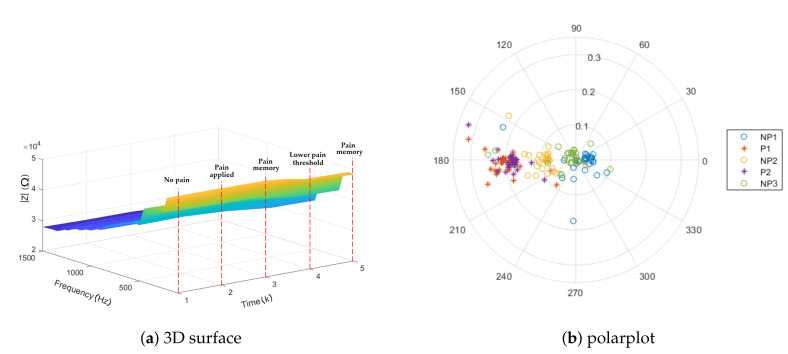
Impedance representations for one volunteer (NP denotes no pain and P denotes pain).

The response to a mechanical pressure induced pain is analyzed, using the protocol proposed in Section 3.2. The 3D representation of the absolute value of impedance is depicted in Figure 7a for one subject. The result indicates different impedance values for the moments of pain applied versus the baseline (first no pain interval). However, when the application of pain has stopped, the small increase in the impedance values can show the pain memory of the tissue, followed by a lower threshold for the second interval of pain application. The surface generated by the analysis of the impedance in function of frequency and time illustrates the change in frequencies. The same trend is depicted in Figure 7b, where the normalized values of impedance for NP2 are localized in between P1 and P2 as well as NP1 and NP3 on the polar plot. Finally, this result suggests that Anspec-PRO can detect changes in impedance in the presence and absence of pain, while in-depth specific frequency analysis is required.

### 3.4. Repeatability and Impedance Dependence

To investigate the device repeatability, another protocol is performed in this work for impedance analysis, but using thermal pain stimulus this time. The multisine (i.e., in the specific frequency range), the location of the electrodes (i.e., palmar skin) and the data analysis for obtaining complex impedance are the same as mentioned in Section 3.3 for the mechanical-induced pain protocol.

**Figure 8:**
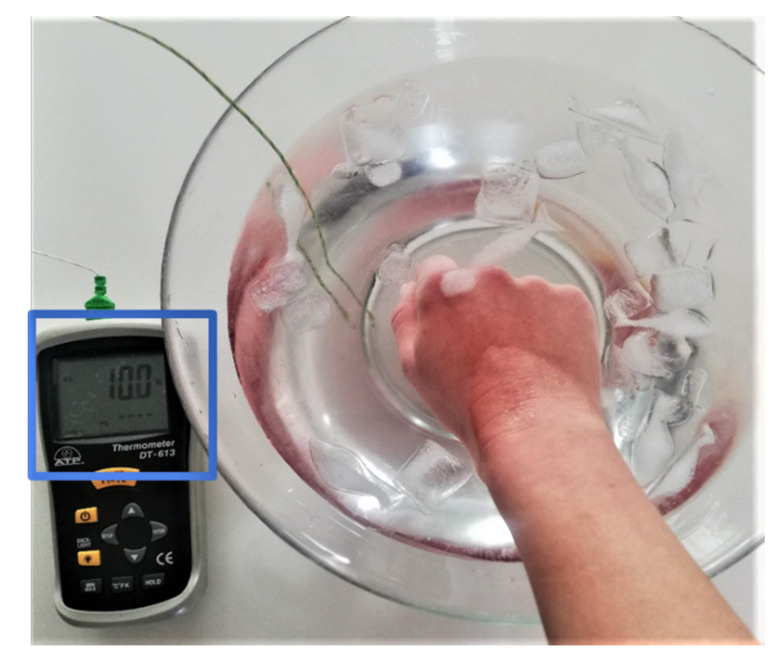
The set-up for the thermal protocol using hand immersion in iced-cold water at 10 ${}^{\circ }$C.

Thirteen different volunteers (i.e., from the development team) are measured for 14 min while immersing one of their hand in a recipient with ice-cold water at 10 ${}^{\circ }$C in an alternating manner (see Figure 8. The biometric data of the subjects included in the study are presented in Table 1. The timeline of the protocol is as follows:

- Minutes 1–2: The subject is in the nominal state, without applying any pain stimulation (NP1).
- Minutes 3–5: Thermal pain stimulation is produced by hand immersion in cold water (P1, P2, and P3).
- Minutes 6–8: The pain stimulation is ceased (NP2).
- Minutes 9–11: Thermal pain stimulation is continuously produced as earlier (P4, P5, and P6).
- Minutes 12–14: The pain stimulation is ceased (NP3).

**Figure 9:**
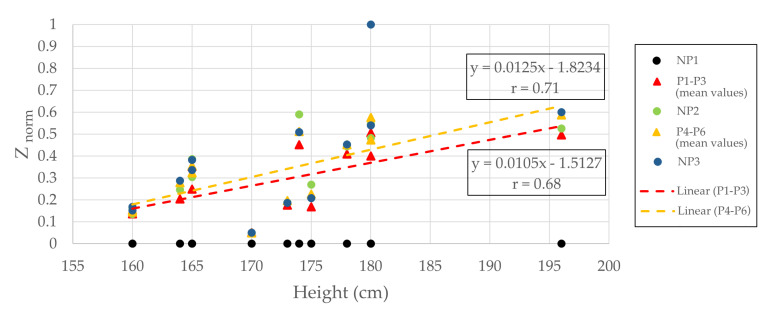
The normalized impedance against height for 13 volunteers.

The absolute values obtained for the impedance are in the same magnitude as for the mechanical-induced pain protocol, but on different volunteers. As observed in Figure 9, there is a linear dependence between the normalized values of the absolute impedance and height (with correlation coefficients r=0.68 and r=0.71). The results are in agreement with what was expected, as the impedance value is related to the tissue volume of a conductor to which the one current-carrying electrode is connected. Consequently, the overall body height of the participant is highly influencing the impedance measurements.

**Figure 10:**
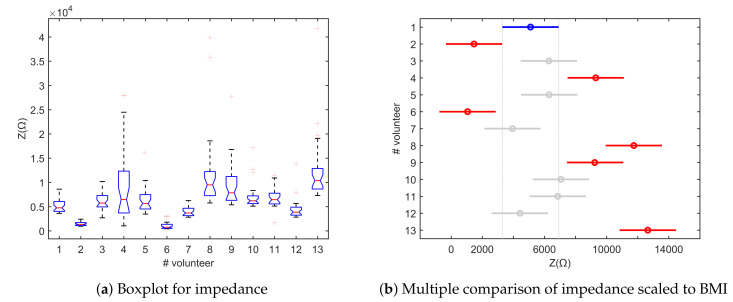
Statistical analysis of measured bioimpedance for all volunteers during thermal test.

**Figure 11:**
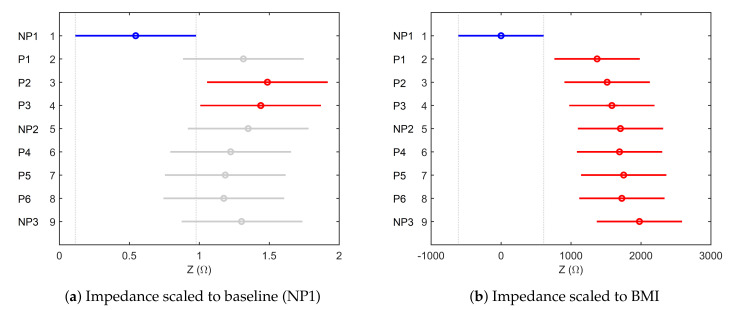
Multiple comparison tests of measured bioimpedance for all intervals during thermal test.

The results of multiple comparison tests and of one-way analysis of variance between volunteers are depicted in Figure 10. The performed statistical analysis in Figure 10a identifies a significant variation between volunteers for the impedance measurements. For the impedance values scaled to the BMI of each volunteer, Figure 10b shows six volunteers (red) significantly different from the comparison group, i.e., Volunteer 1 (blue) and the similar volunteers from the same group (grey). Looking into the multiple comparison between the intervals with pain and no pain stimulus from the thermal protocol, Figure 11a illustrates two intervals significantly different from the others: P2 and P3. This behavior can be explained by the fact the P1 is the first pain interval that still makes the transition between the state of no pain and the stimulation caused by the cold temperature during P2 and P3. For the second period of stimulation (P4–P6), adaptation and numbness occur. Once scaled to BMI, the impedance means show no difference between the intervals in Figure 11b.

### 3.5. Spectrogram Representation

**Figure 12:**
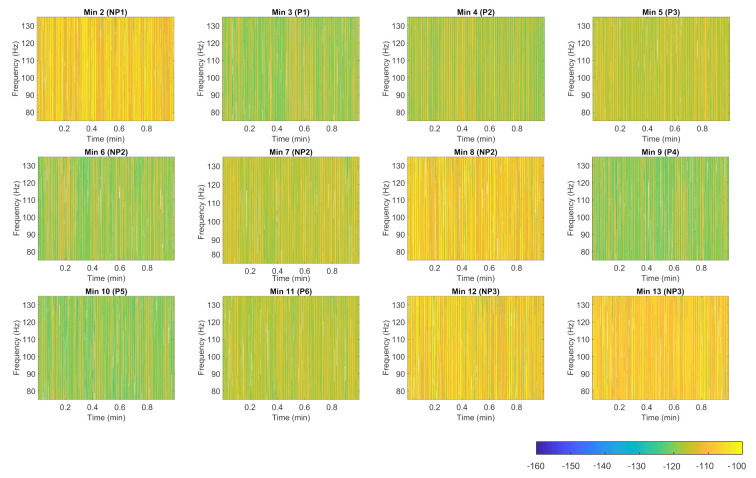
3D representation (spectrogram) of impedance signal for one volunteer measured during the thermal pain protocol (NP denotes no pain and P denotes pain).

The spectrogram representation of the impedance that exhibits the frequency range delivered by Anspec-PRO is further proposed. The time–frequency display may be beneficial in providing useful characteristics of the pain process transfer function. Using short-time Fourier transform, the one-sided modified periodogram estimate of the PSD (power spectral density) or power spectrum of each segment is computed. The spectrogram for one volunteer tested with the thermal protocol, described in Section 3.4, is depicted in Figure 12. For every minute, it displays the spectrum of impedance signal around the excited frequency of 100 Hz in which the power/frequency (dB/Hz) over time is color coded. Starting from the moment of applying pain, the power of bioimpedance is decreasing compared with the baseline moment of no pain (i.e., Minute 2). However, similar behavior is expressed during intermediary minutes of pain absence (i.e., Minutes 6 and 7). Here, the physiological effect that occurs is pain memory, already found in other studies using Anspec-PRO [18,19]. The second interval of induced thermal-pain (i.e., Minute 9) starts once again with visible differences from the last minute of no pain (i.e., Minute 8). The results show different spectrograms of the impedance for pain and no pain minutes, concluding that in-depth analysis of the acquired power spectrum will be able to classify these moments.

Here, we show the utility of the simple time–frequency representation—the spectrogram—in giving probably a more comprehensive yet still intuitive means of visualizing the electrical human response to painful stimulus. However, in-depth analysis on visualization and window properties that affect the spectrogram appearance, as well as more measurements, need to be tackled in the future work. However, this analysis is not the subject of the current paper.

### 3.6. Bioimpedance Modeling

Following the experimental studies, bioimpedance modeling is implied, where fractional-order values are appropriate for model identification (because of the constant phase and slope of the magnitude) [41]. Then, the behavioral derived electrical equivalent circuit model of the skin allows connecting the impedance measurements to the physiological processes. Using an appropriate circuit model to fit the experimental data, the obtaining parameters’ values are further analyzed for the pain–no pain situations. Recent works also discuss fractional-order circuits in various forms [42,43,44,45].

**Figure 13:**
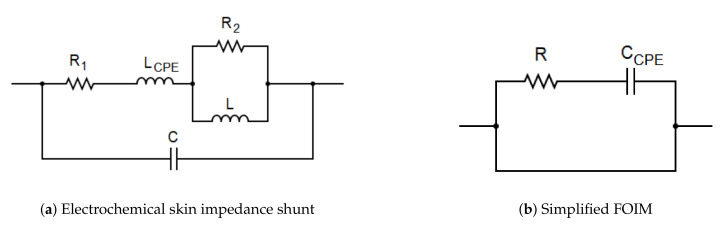
Structure of fractional impedance models proposed.

Originating our prior work on modeling nociception stimulation with fractional-order impedance models (FOIM) [46,47], two FOIM models are then adapted and proposed in their electrical analogs in Figure 13. For the individuals undergoing the thermal tests, the fitting of the proposed models is done onto intervals with pain stimuli and without.

Therefore, the electrical impedance of the electrochemistry skin impedance shunt model presented in Figure 13a is expressed from the frequency response in the complex variable s=σ*jω, as follows:

(11) $$Z_{FOIM}\left(s\right)=\frac{1}{R_{1}+L_{CPE}s^{\alpha }+\frac{1}{R_{2}}+\frac{1}{Ls}}+\frac{1}{Cs}$$

where α is a fractional parameter (0≤α≤1) that defines the frequency behavior of the bioimpedance dispersion.

Different non-integer order values from the FOIM model (Equation (11)) are then identified for one subject in Table 2.

The second model proposed is a FOIM model composed of fewer elements, i.e., a resistance (R) and a constant phase element (CPE) at a fractional order:

(12) $$Z_{FOIM-CPE}\left(s\right)=R+\frac{1}{C_{CPE}s^{\alpha }}$$

with the identified parameters for one subject in Table 3.

**Figure 14:**
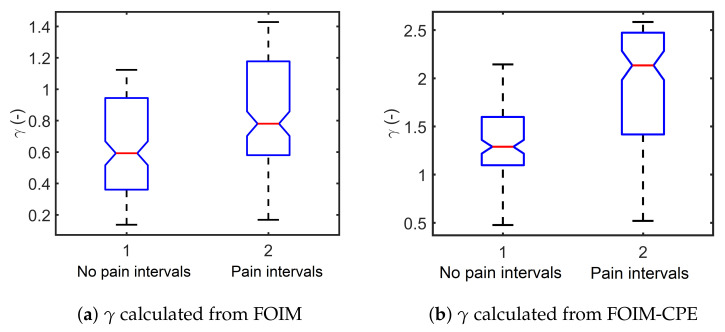
Boxplot of the calculated histeresivity from the frequency response data of all volunteers.

To compare the extracted parameters of stimulated and non-stimulated skin tissues, individual differences can be seen for values characterizing pain (P1) versus no pain intervals (NP2). However, the statistical analysis of impedance variation between pain (P1) and no pain intervals (P2) for all the volunteers included in the study did not show a significant difference (p>0.05). The only exception is the parameter corresponding to the CPE impedance (1/C) from Equation (12), which was found to be significantly bigger for no pain intervals (p<0.05). One step further is to look at the capacitance term in Equation (12), which can be split into real and imaginary parts as follows:

(13) $$G\left(s\right)=\frac{1}{Cs^{\alpha }}cos\left(\alpha \frac{\pi }{2}\right),\;H\left(s\right)=\frac{1}{Cs^{\alpha }}sin\left(\alpha \frac{\pi }{2}\right)$$

with the ratio $\gamma =\frac{Gs}{Hs}$ proposed in [47] as a relation to hysteresivity that describes the dynamics of the tissue in response to sinusoidal behavior. Figure 14 depicts the analysis of variance of γ, resulting in significant higher hysteresivity met in tissue during painful stimulus. Then, a similar trend is followed for γ variation obtained from both models.

From this identification procedure done on all volunteers, it was observed that a significant intra-patient variability between pain and no pain characterizing parameters exists. Nevertheless, the inter-patient variability is too high, so a significant difference between all pain and no pain intervals from all volunteers cannot be obtained. From here, one can deduce that Anspec-PRO needs calibration per person, as one population model that can estimate generalized impedance values is not going to fit everyone. The solution is a personalized adaptive approach, opposed to the commercial pain monitors that usually give a pain index based on population models.

**Figure 15:**
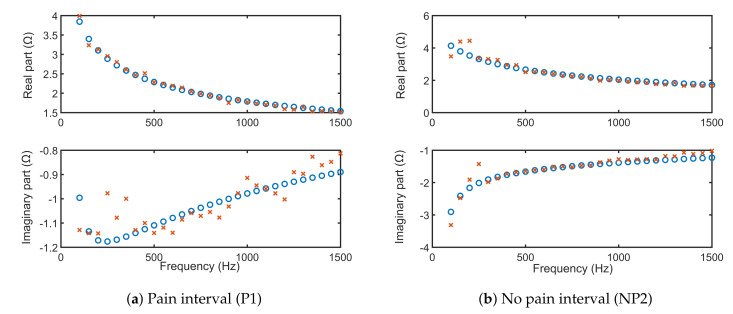
Identified FOIM model for the frequency response data of one volunteer.

**Figure 16:**
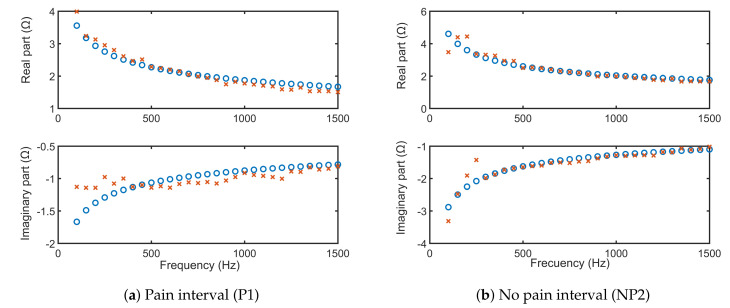
Identified FOIM-CPE model for the frequency response data of the same volunteer.

For only one volunteer, the fitting of the FOIM models from Equations (11) and (12) onto the frequency response complex impedance data are depicted in Figure 15 and Figure 16, respectively. The simulations using extracted FOIM parameters are given as blue circles, while data are represented in red. Both models applied to characterize the skin electrical impedance show a good fit with the frequency dependent tissue impedance. This further validates that the FOIM model is appropriate to be used for fitting collected bioimpedance datasets.

### 3.7. Other Features

Both generations of Anspec-PRO device have already been used in several studies and a summary of the findings is given below.

- To investigate temperature tolerance of volunteers, the following hypotheses were tested on first Anspec-PRO: (i) the bioimpedance is increasing in time when the volunteer has one of his/her hands immersed in ice-cold water due to the new environment change and impact with ice-cold water; and (ii) the impedance of extracellular fluid is dependent on each individual after the threshold of pain is reached. The outcome of these measurements indicates that the device can successfully detect thermal pain. More details about this analysis can be found in [19].
- The authors also investigated detection of nociceptor stimulation followed by related tissue memory effects, using first Anspec-PRO. The method and model indicate that nociceptor stimulation perceived as pain in awake healthy volunteers is non-invasively detected. The existence of a memory effect is proven from measured data [18].
- Spectrographic analysis of the skin impedance measured with second Anspec-PRO device was approached to obtain a wider view towards the signal [20].
- Model identification was performed based on parametric methods. For identification purposes the multi-frequency voltage and corresponding current signals (multisine input–output) are used. More details about it can be found in [48].

## 4. Discussion

Pain is a subjective sensation with biological origins that depends strongly on the type of nociception phenomena [49]. Different from pain as a feeling, nociception denotes the complex sensory process that provides signals for pain triggering [50]. To this extent, characteristic pain indicators can be found in both mental stress effects (e.g., emotional sweating) and nerve endings transduction and transmission (e.g., nociceptors). As electrodermal phenomena are correlated with the psychological arousal, the human sweat is commonly measured through the skin conductance level or the responses originated from the activation of the sympathetic nervous system [8,51]. Moreover, when the nociception is initiated, the peripheral nociceptors fire, generating action potentials. They represent the depolarization of nociceptors (i.e., neuronal membranes), which can result from released substances and ion-gated channels activation by different stimuli [50]. This trans-membrane potential is dependent on the concentration gradients of ions across the membrane that determines different electrophysical properties and can be sensed through bioimpedance.

This paper underlines the important feature of the newly developed prototype Anspec-PRO, namely the possibility to describe the complex impedance in both time- and frequency-related characteristics. The benefit introduced by this sensor is the volume of data collected and the access to different post-processing methods available. Due to the customizable input signal, the desirable optimal extraction of dynamic process information is possible, with the aim of model identification of the excited process. Unlike commercial pain monitors, the promising results presented in this paper on FOIM identification show the opportunity of going in the research direction of personalized medicine, specifically obtaining a pain descriptor calibrated per each patient, and not based on population models. Further in-depth time–frequency analysis of the skin impedance to be correlated to pain levels is required for clinical relevance, using the proposed Anspec-PRO for acquiring individualized complex information.

It is shown that fractional-order impedance models are natural solutions to pain pathway [18]. This paper presents results on the ability of two proposed fractional-order models to catch the variations of the impedance measured during painful excitation and in absence of it. Modeling such phenomena in terms of ordinary differential equations of integer order typically leads to unwieldy sets of equations that appear contrived. On the other hand, differential equations of fractional order tend to naturally capture intrinsic phenomena in complex dynamical systems [52,53]. Their lumped equivalent models are parsimonious representations with few free parameters and have a seemingly natural ability to mimic nature [54,55,56].

The added value of our prototype over other pain sensors consists on allowing to send a range of frequencies to excite the system for pain level evaluation in patients undergoing anesthesia or ICU recovery. For the Anspec-PRO device, the frequency range for skin excitation proven in this work to successfully capture pain induced changes in bioimpedance is [10;1500] Hz. Comparatively, other future research users have the possibility to personalize the input signal.

While the impedance data as well as the methodology developed in this work have proven the tested hypothesis in terms of nociception detection and modeling, a limitation of this study is that the obtained impedance values are yet to be integrated in a personalized index for pain. Perhaps, the small number of subjects included in the self-experimental evaluation can be considered also a limitation. In addition, one could evaluate the nerve endings from the epidermis and dermis layers of skin that respond to painful stimulation; nevertheless, such an invasive method is not directly useful in our endeavor.

The already available results indicate that the developed prototype is able to capture the signaling pathways and deliver an objective measure for pain. A clinical trial was conducted in patients recovering from anesthesia, and the results show a good performance for the prototype to identify changes in pain pathways by measuring the skin impedance [20]. As the aim of this paper is to illustrate the development methodology of our prototype, the existing prior results with more details about the previous work can be found in Section 3.7.

## 5. Conclusions and Perspectives

This paper describes the development and validation of a bioimpedance sensor for time–frequency analysis of pain phenomena. The Anspec-PRO device uses off-the-shelf accessible tools and signal processing algorithms explored at their full potential. The device is non-invasive and portable in its design, while versatile to user-defined testing conditions. The device offers access to raw data for post-processing analysis and is easily adjusted to the design of an experiment (e.g., different protocols and different simulations for pain). The input signal can be altered by the user without the need for additional hardware, which avoids high experimental costs.

As for the next steps, an individualized index of pain level from Anspec-PRO is envisaged for use in monitoring communicative and anesthetized patients. One other phase is the in-depth spectrographic analysis, by using deep learning algorithms for extracting the optimal features that correlate best with the NRS given by the patient (for awake patients) or with BIS (for anesthetized patients). Along with the post-processing, the prototype Anspec-PRO is currently involved in a randomized controlled trial for intraoperative nociception monitoring during total intravenous anesthesia in University Hospital Ghent, Belgium, approved by the Ethics Committee (BC-08020) and by the Federal Agency for Medicines and Health Products Brussels (FAGG/80M0840).

## Figures and Tables

**Table 1:** Biometric characteristics data for the subjects included in this study (BMI = Body Mass Index).

# Volunteer	1	2	3	4	5	6	7	8	9	10	11	12	13
Gender	M	F	F	M	F	M	M	F	F	M	M	M	M
Age (Year)	24	22	40	24	24	22	24	27	27	25	33	23	23
Weight (kg)	57	110	102	55	53	65	67	71	68	63	65	70	76
Height (cm)	164	160	174	160	165	170	173	180	178	180	165	175	196
Fat (%)	14.75	51.22	44.22	15.10	23.48	15.84	16.18	27.1	26.56	20.04	19.44	16.51	12.83
BMI	21.19	42.96	33.69	21.48	19.46	22.49	22.38	21.91	21.46	19.44	23.87	22.85	19.78

**Table 2:** Parameters obtained from FOIM model (Equation (11)) identification.

Interval	$R_{1}$	$L_{\mathrm{CPE}}$	α	$R_{2}$	*L*	*C*
P1	0.099	0.008	0.430	29.075	0.062	131.7889
NP2	0.155	0.002	0.527	102.276	2904.545	0.0009

**Table 3:** Parameters obtained from FOIM-CPE model (Equation (12)) identification.

Interval	*R*	$1/C_{\mathrm{CPE}}$	α
P1	2.683 × 10${}^{-10}$	23.732	0.279
NP2	5.437 × 10${}^{-10}$	53.857	0.356

**Figure A1:**
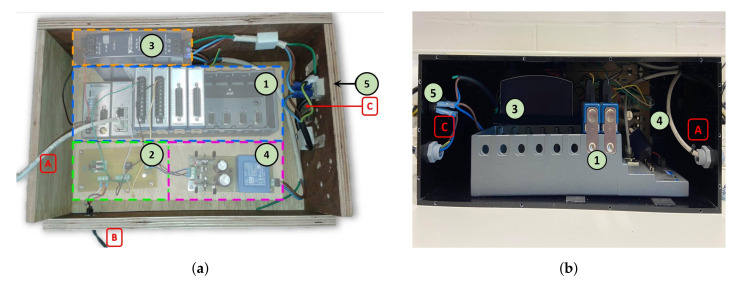
Proposed in-house prototype and equipment for bioimpedance measurements: Anspec-PRO. Two generations are presented: (**a**) the first generation (wooden box) ((1) cRIO; (2) signal conditioning circuit; (3) power supply of cRIO; (4) power supply for (2); (5) on/off-switch; (A) Ethernet cable; (B) electrode connector; and (C) power cable); and (**b**) the second generation with the optimized design for use in clinical trials that contains the same components as the first version.

**Figure A2:**
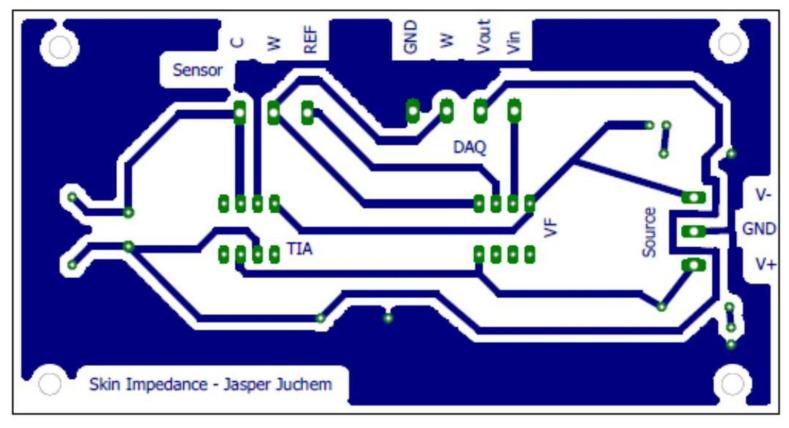
The printed circuit board layout for the VB, TIA, and its connectors.

**Figure A3:**
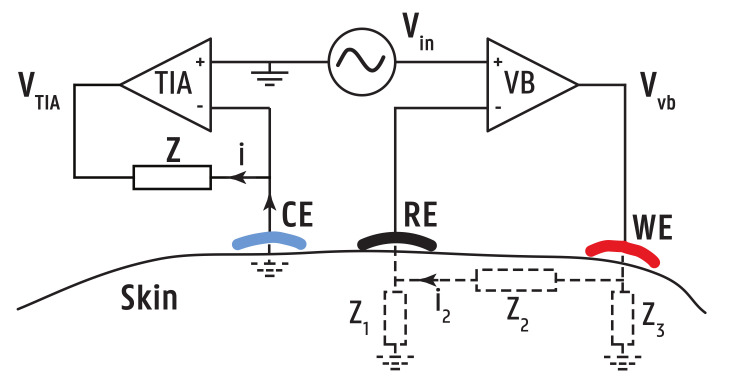
Block diagram for the signal conditioning circuit with Voltage Buffer (VB) and TransImpedance Amplifier (TIA). The dotted line represents a simplified representation of the electrical equivalent network of the skin. The three electrodes are indicated with Counter (CE), Reference (RE), and Working (WE) electrode.

**Figure A4:**
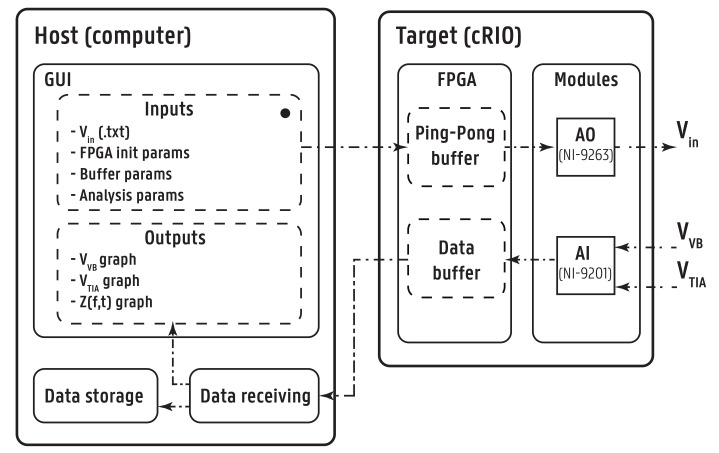
Software architecture and data flow indicated by the arrows.

**Figure A5:**
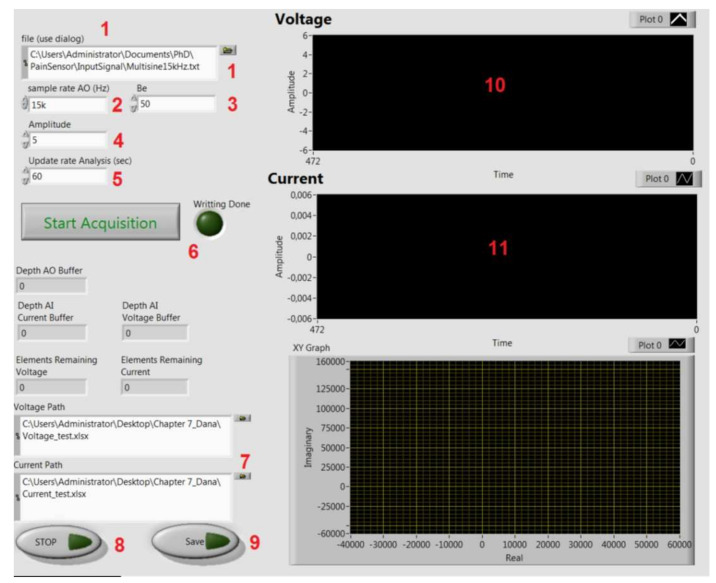
Graphical user interface on the laptop that connects to Anspec-PRO device.

## Abbreviations

The following abbreviations are used in this manuscript:

AI	Analog Input
ADC	Analog-to-Digital Converter
AO	Analog Output
BIS	BioImpedance Spectroscopy
CE	Counter Electrode
cRIO	compactRIO
DAC	Digital-to-Analog Converter
EDA	Electrodermal Activity
FFT	Fast Fourier Transform
FRF	Frequency Response Function
FPGA	Field-Programmable Gate Array
GUI	Graphical User Interface
MSRE	Mean Square Root Error
Op Amp	Operational Amplifier
PSD	Power Spectra Density
RC	Circuit with both a resistor (R) and a capacitor (C)
RE	Reference Electrode
RMS	Root Mean Square
SC	Stratum Corneum
TIA	TransImpedance Amplifier
VB	Voltage Buffer
VS	Viable Skin
WE	Working Electrode

## Appendix A. Frequency Ranges

Different purposes for impedance measurements have decided the frequency range of tissue excitation for each application, as depicted in Table A1.

**Table A1:** Frequencies used for impedance measurements.

Level	Frequency Range	Reference
Cells	100 Hz to 1 MHz	[32]
	10–1 to 105 Hz	[33]
Skin	30 Hz–10 MHz	[57]
	20, 60, 100, 140, 180, 220 Hz	[58]
	5 kHz to 1 MHz	[59]
	10 Hz to 100 kHz	[60]
Muscle	0.02, 25.02, 50.02, 1000.02, 3000.02, and 5000.02 kHz	[57]
Entire body	10 Hz, 1 kHz, 100 kHz, 10 MHz, 1 GHz, and 100 GHz	[35]
Bioelectrical impedance analysis	5 to 1000 kHz	[36]
	100 Hz to 100 kHz	[37]
	4–1000 Hz	[61]
	32 to 992 kHz	[62]
	30 Hz–10 MHz	[57]

## Appendix B. Hardware Components

The prototype Anspec-PRO was firstly designed in a wooden box for tests in our laboratory [18,19], as the first generation is depicted in Figure A1a. For use in clinical trials [20], a second generation was developed, displayed in Figure A1b.

The hardware architecture consists of several parts that are illustrated in Figure A1:

1. the data-acquisition unit (1) National Instruments’™ *compactRIO* connected to its power supply (3); and
2. the signal conditioning circuit (2), also connected to its power supply (4).

A laptop is used to capture, save, and display measured data. An Ethernet cable (A) provides the communication between the device and the laptop, over a local network. The device is connected to the electrodes cable (B) and to the power cable (C). The electrodes and electrode connectors are CE-marked (according to MDD93/42/EEC).

The prototype follows a modular design scheme (see Figure A1a):

- Data-acquisition is done with the embedded controller CompactRIO-9074 (cRIO) from National Instruments (Austin, Texas), which has a Field-Programmable Gate Array (FPGA) chip (Xilinx’ FPGA Spartan-3 1M), particularly useful for setups where a minimal robustness must be ensured for sending and acquiring signals in real-time. The FPGA is programmed within LabVIEW software. The National Instruments PS-15 Power Supply is used to power two C-series I/O modules:
  - *Analog-to-Digital* (ADC) converter as Analog Input (*NI-9201*): voltage input signals are scanned, buffered, conditioned, and then sampled by a single ADC.
  - *Digital-to-Analog* (DAC) converter as Analog Output (*NI-9263*): each channel of the Analog Output module has a DAC that produces a voltage signal.
  These are stand-alone data acquisition units and are used for converting the physical signal (analog) into a sequence of bits (digital signal) or vice versa.
- Signal conditioning circuit is an interface between the computer and the electrodes, mitigating unwanted effects, such as noise or AC coupling. The signals coming from and going to National Instruments’ cRIO need to be conditioned before reaching the skin. This is done with a circuit containing operational amplifiers, which are connected to a DC power source with the voltage limited to ±10 V. The circuit encompasses:
  - *Operational Amplifiers’ voltage source*, in-house created for acquiring at least a +10 VDC and −10 VDC output compared to a common ground, as required by the I/O module manuals. An artificial Ground (GND) is created by the means of a galvanic separator, a transformer. However, to achieve the wanted supply voltages, a low drop-out voltage regulator is used to stabilize the output voltages. A rail with a positive voltage (MC7812) and with a negative voltage (MC7912), compared to this GND, are supplied to the voltage regulators, with the output voltage ±12 V.
  - *Voltage Buffer* (VB), which assures a correct voltage at the output of the circuit, being used just for transferring the voltage from the circuit to the skin. The active component is an Operational Amplifier (Op Amp) (*Texas Instruments™ MC1458P*). In a classic voltage buffer design, the output of the Op Amp is connected to the inverting supply, to achieve negative feedback. However, in this case, the voltage signal is first sent through a filter, namely the skin. The output signal is limited to ±15 V and ±25 mA.
  - *TransImpedance Amplifier* (TIA) circuit consists of an Op Amp (*Analog Devices™ ADA4077*), which, together with a resistor-capacitor circuit, allow transforming the current into a voltage. This process is motivated by the fact that ADC AI connected to cRIO can acquire only voltages, but current is also required for impedance calculations. However, by placing a resistance in the network, the current can be calculated from the measured voltage, based on Ohm’s law (v=R×i).

Device components are listed in Table A2, followed by specifications detailed in Table A3.

**Table A2:** Electrical components of the following modules: (cRIO) NI-compactRIO; (S) Op Amp voltage source; (VB) Voltage Buffer; and (TIA) Transimpedance Amplifier.

Module	Component	Manufacturer	Nr
cRIO	cRIO-9074	National Instruments	1
cRIO	NI-9201	National Instruments	1
cRIO	NI-9263	National Instruments	1
cRIO	PS-125	National Instruments	1
cRIO	Ethernet cable (5E - patched)	/	2
S	PCB	EELAB	1
S	2way PCB Terminal Block	Phoenix	1
S	Transformer 15 V	Block	1
S	Diodes −400 V	ON Semiconductor	6
S	Electrolytic Capacitor (3300 nF–35 V)	United Chemi-Con	2
S	Voltage Regulator (+12 V/1 A) (MC7812)	ON Semiconductor	1
S	Voltage Regulator (−12 V/1 A) (MC7912)	ON Semiconductor	1
S	Electrolytic Capacitor (100 μF–25 V)	Nichicon	2
S	Film Capacitor (0.1 μF–63 V PET)	Kemet	2
S	Heat Sink TO-220	AAVID-Thermalloy	2
S/VB	3way PCB Terminal Block	Phoenix	3
VB	Op Amp MC1458P	Texas Instruments	1
VB	4way PCB Terminal Block	Phoenix	1
VB	Audio Connector (3 contacts, Jack 3.5)	Pro Signal	1
VB	Sensor Cable (3 electrodes, Jack 3.5)	/	1
VB/TIA	PCB	Own Design	1
TIA	Op Amp ADA4077-2	Analog Devices	1
TIA	Resistance (1000 Ω–5%)	Velleman	1
TIA	Ceramic Capacitor (10 pF–50 V)	Kemet	1

**Table A3:** Key specifications.

Component	Electronic Specifications
ADC *cRIO NI-9201*	Input range: ±10 V; 500 kS/s; ADC resolution: 12-Bit; Measurement voltage: ±10.3 V; Overvoltage protection ±100 V.
DAC *cRIO NI-9263*	Output range: ±10 V; 100 kS/s/ch Simultaneous; DAC resolution: 16-Bit; Current drive: ±1 mA per channel maximum; Overvoltage protection: ±30 V.
NI LabVIEW FPGA Module	Loop rate of executable control algorithms up to 300 MHz.
Power Supply NI PS-15	AC 100–120/200–240 V Auto-select input; The output terminals provide 24–28 VDC with 5 A of current.
TransImpedance Amplifier *ADA4077-2*	Input Voltage Range: ±15 V; Output voltage range: 14 V; Output Current: ±10 mA; Gain Bandwidth: 3.6 MHz.
Voltage Buffer *MC1458*	Input Voltage: ±15 V; Supply voltage: 15 V; Maximum-output-swing bandwidth (closed loop): 14 kHz; Unity Gain Bandwidth: 1 MHz; Short-circuit output current: ±25 mA; Supply current (both amplifiers): 3.4 mA.

The printed circuit board layout is presented in Figure A2 with a magnification of 100%.

The conditioning circuit for a three-electrode measuring technique is designed using a standard electronics circuit described in [24]. DAC module generates an alternating electric voltage of known amplitude to be injected into the human body through one electrode, after VB filtering. Thus, the multisine signal $V_{vb}$ filtered with the VB configuration is applied on the skin, via the working electrode (WE). This voltage induces a corresponding current in the circuit related to the impedance of the human body. The current pathways are from WE to the counter electrode (CE). This CE is grounded through the TIA, while no current flows into the reference electrode (RE) due to the high input resistance of Op Amp. All the current *i* is drawn towards the CE, which closes the circuit. The impedance *Z* transforms the current into a voltage that is proportional to the current, i.e., $V_{TIA}\left(s\right)=Z\left(s\right)\cdot i\left(s\right)$. By means of RE located in the current path, the TIA subsystem measures the voltage generated by the circulation of the current. To obtain both the voltage and current signal in time, $V_{TIA}\left(t\right)$, and $V_{vb}\left(t\right)$ are acquired simultaneously. A schematic representation of the interface is given in Figure A3.

Ideally, the RE electrode is placed very closely to CE electrode. In [24], a better electrode design is proposed based on this observation. The electrode consists of three concentric rings. The inner ring is a circular CE, the middle ring of non-conductive material is used to separate the inner circle from the outer ring, which consists of a conductive ring of gel to act as the RE. By using the three-electrode configuration, one achieves a quantification of the impedance underneath the CE. The upper skin layer, the *Stratum Corneum* (SC), has a much higher resistivity than the underlying viable skin (VS) layer. As the skin is a volume conductor, the current will be spread over the volume, bringing into attention the current density *J* [A/m${}^{3}$]. However, the current flow can be simplified due to the difference in resistivity and the fact that current will follow the path with the least resistance. The current will take the shortest path through the SC towards the less-resistive VS. Next, it will flow until it reaches the CE. The current will flow to the CE electrode through the SC, again taking the shortest path. Because of the lower resistivity, the impedance of the SC right underneath the CE will dominate the reading of impedance.

For safety reasons, the device’s voltage and current are limited to protect the patient/volunteer. The protection is in accordance with the European Directive for Medical Devices [63].

## Appendix C. Software Architecture

The software architecture is twofold: the host computer software and the target (cRIO) FPGA software, as given in Figure A4. The LabVIEW software package provided by National Instruments graphical programming was used for deployment.

The flowchart for software sequence is detailed next.

1. Before starting a new acquisition, all parameters are initialized and the graphs are cleared in the host program.
2. The user manually introduces the analysis specific parameters and the path that indicates the location of the .txt file containing the input/excitatory multisine signal.
3. If the parameters are correctly assigned, the .txt file is read and converted into an array with double precision numbers.
4. All buffers are initialized and sufficient memory is allocated in order to avoid data overflow.
5. The host sends the parameters and the signal array to FPGA target with the help of a *First-In-First-Out (FIFO) Host to Target—Direct Memory Access (DMA)* buffer, called Analog Output (AO). DMA makes it possible to transfer data from one hardware subsystem to another in real-time. The data are saved into another buffer AOH1 which is a *Target-Scoped Block Memory FIFO* buffer, with the same specifications of AO. Another buffer AOH2 is identical to buffer AOH1, hence both allow the ping-pong procedure explained below for the AO loop.
6. If the host buffer is emptied and all data are copied on the target, the target software is ready for starting the data acquisition. The user receives a green-light in the graphical user interface (GUI) to start the data acquisition from the patient.
7. Once the user starts the data acquisition, on the FPGA software, there are two parallel *while* loops: the *Analog Output (AO)* loop and the *Analog Input (AI)* loop:
  7.1. In the AO loop, the input array $V_{in}$ enters the *Ping-Pong Buffer*. The array elements ping-pong between two buffers, one active and one passive.
  7.2. With every sampling period $T_{s}$, the active buffer is emptied element-wise and each value is sent to the output port (DAC module), while also saved in the passive buffer. When one buffer is filled, they interchange between being active and passive, thus creating a continuous periodic signal by repeatedly sending to the electrodes the same array of numerical values $V_{in}$. This particular implementation architecture enables to continuously iterate through an array on the FPGA chip in delay-free real-time.
  7.3. In parallel, the I/O hardware module measures the voltages from electrodes. With reference to Figure A3, AI modules acquire the voltage ($V_{VB}$) from WE and the voltage of the output of TIA ($V_{TIA}$), after being transformed from the bio-current by the active circuit implemented in the device.
  7.4. The DAC from AI module transforms the analog signals into digital data.
  7.5. For every sample from ADC, the two voltages, $V_{VB}$ and $V_{TIA}$, are stored in the *Data Buffer*, which is represented by two FIFO buffers: AIVoltage (for $V_{VB}$) and AICurrent (for $V_{TIA}$). Pipeline method is used to guarantee that acquiring the voltage and writing it to the buffer is done simultaneously.
  7.6. The *Data Buffer* is emptied in batches through a UTP cable into the *Data Receiver*, which is a container for all data on the host software.
8. In parallel, a *while* loop on the host side starts reading all elements waiting in the *Data Buffer* (AIVoltage and AICurrent) into the *Data Receiver*. The measured voltage $V_{TIA}$ that describes the current is processed in the host software and the correct equivalent voltage is obtained. The data undergo a 180${}^{\circ }$ phase shift, because the inverting properties of the *op–amp*, and the numerical values are divided by the resistance, which is 1000 Ω.
9. The *while* loop is stopped whenever an error is encountered or when the user presses the Stop button.
10. The user can save the data in a .xlsx file every $T_{s}$ on a specific location on the computer. The parameters $V_{VB}$ and $V_{TIA}$ are plotted in real time in the GUI. The impedance $Z(\omega ,nT_{s})$ is calculated and given in parallel with the data-acquisition and printed in the graphical user interface (GUI).

## Appendix D. User Interface

The host software returns the graphical user interface (GUI) for the measurements and provides the user for the software initialization and parameters definition, followed by visualization in real time of the output signals.

Prior to data acquisition, the host software demands several parameters that are unique for each experiment:

- the input signal $V_{in}$;
- FPGA timer parameters;
- buffer allocation parameters; and
- parameters influencing the data analysis.

To initiate a measurement loop, the user sees the interface presented in Figure A5. The following fields are depicted: (1) file for input signal a priori designed; (2) sampling frequency [Hz]; (3) frequency resolution [Hz]; (4) maximum amplitude of output signal, in order to control saturation of the input signal to the patient (seen on 10); (5) default number of seconds that are saved per data file (60 s); (6) start acquisition button; (7) path for data saving (current and voltage will be recorded in an excel file); (8) stop acquisition button; (9) save data button; and (10 and 11) figures of the input voltage and the output current.
